# Prognostic role of copeptin after stroke: A systematic review and meta-analysis of observational studies

**DOI:** 10.1038/srep11665

**Published:** 2015-06-29

**Authors:** Kyu-Sun Choi, Hyun Jung Kim, Hyoung-Joon Chun, Jae Min Kim, Hyeong-Joong Yi, Jin-Hwan Cheong, Choong-Hyun Kim, Suck-Jun Oh, Yong Ko, Young-Soo Kim, Koang-Hum Bak, Je-Il Ryu, Wonhee Kim, Taeho Lim, Hyeong sik Ahn, Il Min Ahn, Seon-Heui Lee

**Affiliations:** 1 Department of Neurosurgery, College of Medicine, Hanyang University, Seoul, Korea; 2 Department of Preventive Medicine, College of Medicine, Korea University, Seoul, Korea; 3 Department of Emergency Medicine, College of Medicine, Hallym University, Seoul, Korea; 4 Department of Emergency Medicine, College of Medicine, Hanyang University, Seoul, Korea; 5 Department of Literary Arts, Brown University, Rhode Island, USA; 6 Department of Nursing Science, College of Nursing, Gachon University, Incheon, Korea

## Abstract

Copeptin, the C-terminal part of provasopressin, has emerged as a novel prognostic marker after hemorrhagic or ischemic stroke. The aim of this study was to quantitatively assess the prognostic significance of plasma copeptin level on functional outcome and mortality in patients with acute stroke using a meta-analysis of the available evidence. Thirteen relevant studies from 2,746 patients were finally included in our study. An elevated plasma copeptin level was associated with an increased risk of unfavorable outcome and mortality after stroke (OR 1.77; 95% CI, 1.44–2.19 and OR 3.90; 95% CI 3.07–4.95, respectively). The result of the pooled measure on standardized mean difference (SMD) was that plasma copeptin levels were found to be significantly higher in patients who died compared to survivors (SMD 1.70; 95% CI, 1.36–2.03). A stratified analysis by study region showed significant differences in SMD of copeptin, and the heterogeneity among studies was significantly decreased. However, the positive association of copeptin with poor prognosis after stroke was consistent in each stratified analysis. The present meta-analysis suggests that early measurement of plasma copeptin could provide better prognostic information about functional outcome and mortality in patients with acute stroke.

Stroke is a disastrous disease; it is one of the leading causes of death and serious disability worldwide^1^. Mortality after 1 year ranges between 21% and 27%; approximately 15% to 30% of stroke survivors are permanently disabled^1^. It is necessary to evaluate prognostic factors to predict functional outcomes and mortality after stroke, which could be effective in creating therapeutic strategies and improving survival rates. Patient age and stroke severity according to the National Institutes of Health Stroke Scale (NIHSS) score on admission are considered to be independent prognostic factors for survival after stroke. However, these clinical factors are insufficient to predict outcomes at stroke onset for individual patients^2^. Early measurement of molecular biological markers could enable a more accurate estimation of disease severity and patient outcomes and provide acceptable therapeutic intervention targets^3^.

Copeptin, the C-terminal part of provasopressin, is a glycopeptide of 39 amino acids that is stable at room temperature and can be easily measured using automated assays, with results available within 20–60 min^4^,^5^. Copeptin might have a role as a sensitive surrogate marker for AVP release indicating the individual stress response, because arginine-vasopressin (AVP) is a potent synergistic factor of the hypothalamo-pituitary-adrenal axis. Copeptin is significantly elevated in patients with stroke, acute myocardial infarction, heart failure, shock, and traumatic brain injury and has also been proposed to be a prognostic marker for poor clinical outcome and death in these patients^5^,^6^,^7^,^8^,^9^,^10^.

In the current study, we performed a systematic review and meta-analysis of the available evidence in order to quantitatively assess the prognostic value of copeptin for functional outcome and mortality in acute stroke patients.

## Results

### Study selection and characteristics

**Figure 1:**
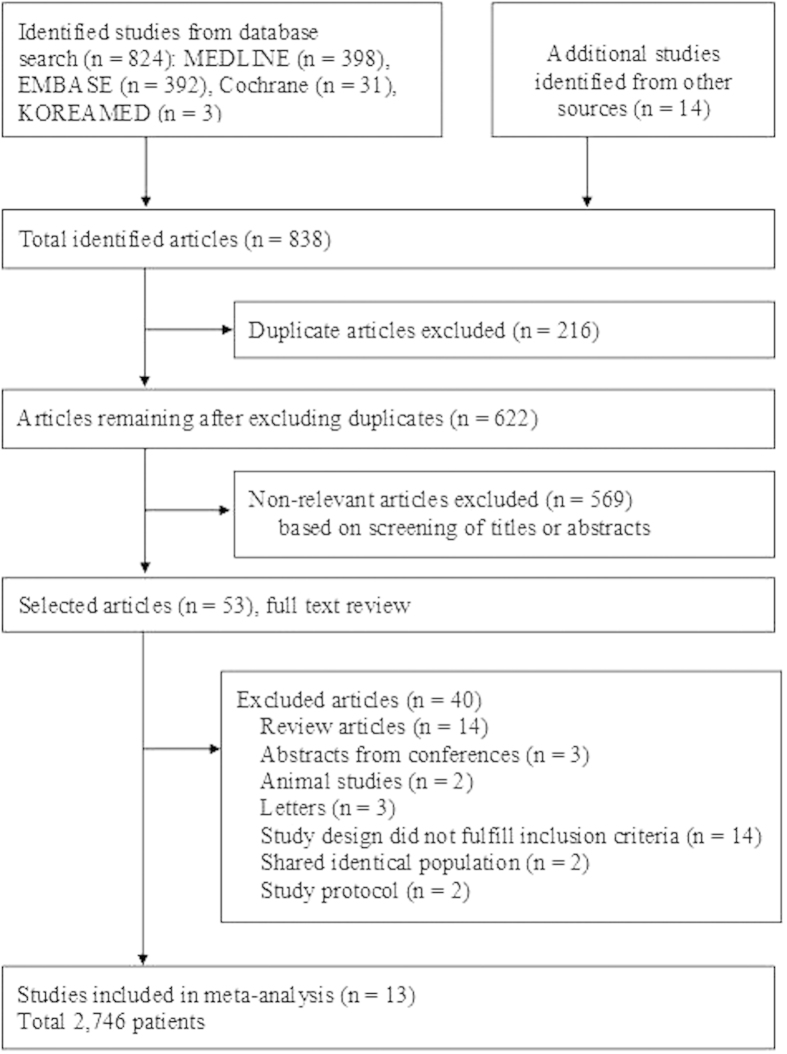
Flow diagram for identification of relevant studies.

The process for identifying eligible studies is shown in Fig. 1. Searches of the databases resulted in 838 articles. A total of 622 studies remained after excluding duplicate articles. Of these, 569 irrelevant publications were excluded based on screening of titles and abstracts. A total of 53 potentially relevant studies were fully reviewed with the full text. Among them, 40 articles were excluded because of the following reasons: review articles (14); abstracts from conferences (3); animal studies (2); letters (3); study design did not fulfill inclusion criteria (14); shared an identical population (2); or study protocol (2). Finally, 2,746 patients in 13 studies met the inclusion criteria and were included in the meta-analysis^8^,^9^,^10^,^11^,^12^,^13^,^14^,^15^,^16^,^17^,^18^,^19^,^20^.

The main characteristics of the 13 eligible publications are shown in Table 1. All included studies were observational. Five studies were about ischemic stroke, while 8 studies were about hemorrhagic stroke. Overall, mortality and functional outcome were obtained from 13 and 12 articles, respectively. Favorable and unfavorable functional outcomes (including mortality) were defined as a modified Rankin Scale score of 0 to 2 and 3 to 6, respectively. Among these studies, 11 studies measured ORs or HRs with 95% CIs from multivariate analysis, while 2 studies only compared the mean value of copeptin between survivors (favorable outcome) and non-survivors (unfavorable outcome)^17^,^19^. ORs and HRs correspond to a 1-unit increase in the explanatory variable; for the log-transformed copeptin values, this corresponds to a 10-fold increase. Of 11 studies, 9 studies tried to control for potential confounding factors by adjusting for known risk factors of poor outcomes after acute stroke such as age, stroke severity, and others^8^,^9^,^10^,^11^,^13^,^14^,^15^,^16^,^18^. Two studies did not adjust for age^12^,^20^.

### Quality of the included studies

Among the 13 included studies, four studies fulfilled all of the quality criteria^8^,^9^,^11^,^15^, and were deemed to be high quality, while the other two studies did not meet two or more criteria^17^,^20^. According to the quality scoring system, 4 of 13 studies were deemed to be low-quality studies^12^,^13^,^17^,^20^. The factors that most affected the quality of the articles were study attrition and the statistical analysis/presentation. Of the total articles, 75% specified that the measurement of biomarkers was blind to clinical data. Details of our assessment of the quality of the included studies are presented in Supplementary Fig. S1 (online).

### Main analysis

**Figure 2:**
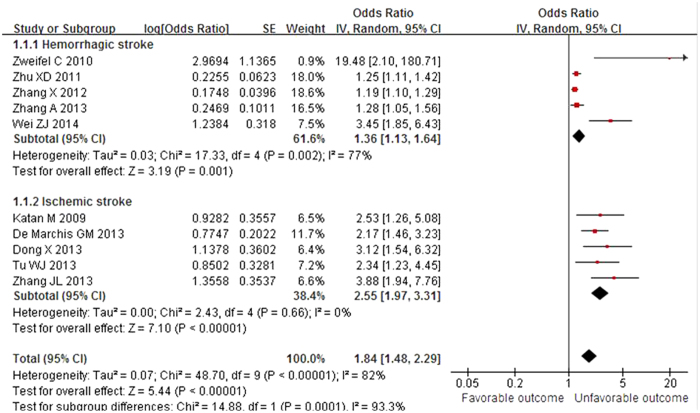
Meta-analysis of relevant studies assessing functional outcome according to stroke subtype (random effects model). Ten observational studies were included. CI, confidence interval; IV, inverse variance; SE, standard error.

**Figure 3:**
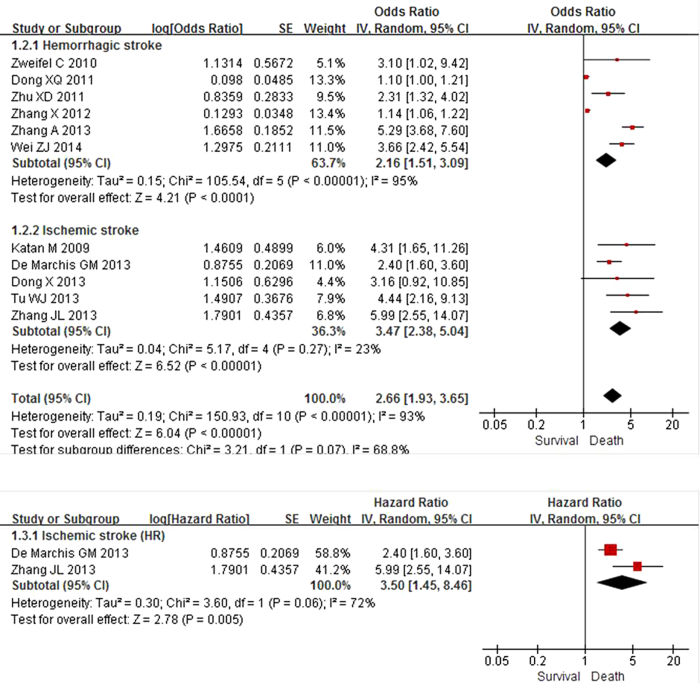
Meta-analysis of relevant studies assessing mortality according to stroke subtype (random effects model). Eleven observational studies were included. CI, confidence interval; HR, hazard ratio; IV, inverse variance; SE, standard error.

Thirteen relevant studies including 2,746 patients were finally included in our study. In our meta-analyses, elevated plasma copeptin level was associated with an increased risk of unfavorable outcome after hemorrhagic stroke (aOR 1.36; 95% CI 1.13–1.64; I^2^ = 77%), ischemic stroke (aOR 2.55; 95% CI 1.97–3.31; I^2^ = 0%), and all types of stroke (aOR 1.84; 95% CI, 1.48–2.29; I^2^ = 82%) (Fig. 2). A more prominent association was found between copeptin level and mortality after hemorrhagic stroke (aOR 2.16; 95% CI 1.51–3.09; I^2^ = 95%), ischemic stroke (aOR 3.47; 95% CI 2.38–5.04; I^2^ = 23%), and all types of stroke (aOR 2.66; 95% CI 1.93–3.65; I^2^ = 93%) (Fig. 3). Among studies reporting HRs based on multivariate analysis, a higher copeptin level was significantly associated with mortality in ischemic stroke patients (HR 3.50; 95% CI 1.45–8.46; I^2^ = 72%)^9^,^15^.

### Subgroup analysis and sources of heterogeneity

We additionally performed subgroup analysis according to the type of stroke (hemorrhagic or ischemic; Figs 2 and 3) and case fatality rate (mortality >35% or lower; see below). Table 2 shows the results of the subgroup meta-analyses. There was a significant difference in the effect of copeptin level on unfavorable outcome with *P* = 0.0001 (aOR 2.55 for ischemic stroke and aOR 1.36 for hemorrhagic stroke). However, the effect of copeptin level on mortality in the hemorrhagic stroke group was not statistically different from that in the ischemic stroke group with *P* = 0.71. The degree of heterogeneity was always lower in the ischemic stroke group compared with that of the hemorrhagic stroke group (I^2^ = 0 vs. 77% for unfavorable outcome; I^2^ = 23% vs. 95% for mortality, respectively). There was a statistically significant difference for the effect of copeptin level on mortality when analyzed according to the case fatality rate in the hemorrhagic stroke group. In studies with mortality over 35% after hemorrhagic stroke with a small sample size (<100) by Dong *et al.*^13^ and Zhang *et al.*^14^, higher copeptin level was less associated with mortality than that of the lower mortality group with *P* < 0.00001 (aOR 1.13; 95% CI 1.07–1.19; I^2^ = 0% and aOR 3.66; 95% CI 2.49–5.36; I^2^ = 53%, respectively). Accordingly, small sample size studies from China in the hemorrhagic stroke group may be the key contributor to between-study heterogeneity^17^,^18^.

When restricted to studies with a large sample size (>100), we found prominent ORs and less heterogeneity (OR 3.64; 95% CI 2.76–4.80; I^2^ = 44%). In the subgroup analysis by study region, a significant association was found in Western studies (OR 2.67; 95% CI 1.87–3.80; I^2^ = 0%). However, high heterogeneities were unresolved when the analysis was restricted to studies including only hemorrhagic stroke, a Chinese population, less than 40% female subjects, blood sampling within 24 hours, or mortality assessment within 3 months. After excluding the 2 articles, the pooled OR of copeptin was 3.61 (95% CI 2.79–4.66; I^2^ = 36%) for mortality after acute stroke. Stratification by the time window of mortality assessment indicated that compared with studies performed at 1 year, studies performed at 3 months exhibited a more significant association between copeptin and mortality (aOR 3.71; 95% CI 2.84–4.86; I^2^ = 30% and aOR 2.30; 95% CI 0.95–5.57; I^2^ = 90%, respectively). In addition, heterogeneities were significantly decreased in each subgroup (Table 2).

### Pooled measure on standardized mean differences

**Figure 4:**
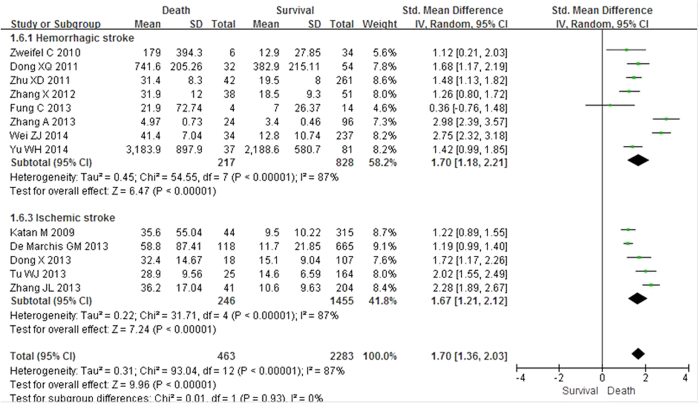
Standardized mean copeptin values in death group and survival group and a pooled estimate (random effects model). Thirteen observational studies were included. CI, confidence interval; IV, inverse variance; SD, standard deviation.

Thirteen studies reported differences in plasma copeptin levels between patients who did not survive and survivors. Since obvious heterogeneity was discovered in the assessment, the random-effects model was used. The result of the pooled measure on standardized mean difference (SMD) was that copeptin levels were found to be significantly higher in patients who died than survivors, demonstrating a positive association with an overall SMD [(mean level in death group – mean level in survival group)/pooled SD] of 1.70 (95% CI 1.36–2.03; I^2^ = 87%; *P* < 0.00001; Fig. 4). In other words, a high copeptin level was associated with increased risk of mortality after stroke. However, significant heterogeneity was present. We evaluated potential sources of heterogeneity in stratified analyses (Table 3). We observed a significant association between higher copeptin level and mortality in both hemorrhagic and ischemic subgroups. A significant association was also found between copeptin levels and mortality in each subgroup (all *P* < 0.05). We observed subgroup differences in studies according to study region. However, associations did not differ substantially by stroke subtype, study size, number of female subjects, time of outcome assessment, time of copeptin measurement or how the mean and SD were obtained (described in published report vs. estimated by recognized formulas).

**Figure 5:**
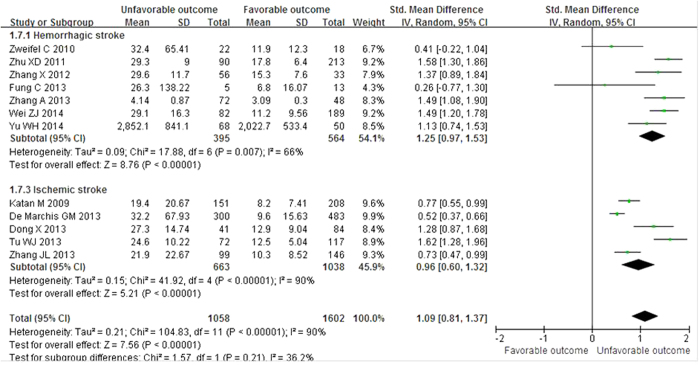
Standardized mean copeptin values in unfavorable and favorable outcome groups and a pooled estimate (random effects model). Twelve observational studies were included. CI, confidence interval; IV, inverse variance; SD, standard deviation.

Twelve studies described plasma copeptin levels according to functional outcome. Patients with unfavorable outcomes demonstrated significantly higher copeptin values than those with favorable outcomes after acute stroke, with a pooled SMD of 1.09 (95% CI 0.81–1.37; I^2^ = 90%; *P* < 0.00001) (Fig. 5). We observed subgroup differences in studies according to study region and how the mean and SD were obtained (Table 3). However, the associations did not differ substantially by the other characteristics as stated above. We were not able to stratify the analysis by the two different hemorrhagic subtypes (ICH or SAH) because only 2 studies for SAH offered relevant available data.

### Sensitivity analysis

A sensitivity analysis was performed by the sequential removal of individual studies one at a time and estimating the overall SMD for the remaining studies. The results of the sensitivity analysis suggested that no single study significantly influenced the overall pooled estimates, indicating that our results were statistically reliable.

## Discussion

This systematic review and meta-analysis demonstrated that increased copeptin levels were significantly associated with unfavorable outcomes and mortality in patients with hemorrhagic or ischemic stroke. Moreover, a significant association of copeptin with poor outcome after stroke was observed in studies which adjusted for important prognostic factors such as age, sex, and stroke severity. These results suggest the possibility that a higher copeptin level is an independent prognostic factor for mortality and unfavorable outcome after stroke irrespective of stroke subtype. Since the prediction of risk for poor outcomes in acute stroke patients remains complicated and mostly depends on underlying conditions or clinical parameters, these findings have important clinical implications.

The prognostic role of copeptin has been reported in various types of acute illness, including hemorrhagic/septic shock, lower respiratory tract infection, heart failure, and acute myocardial infarction^5^,^6^,^8^,^21^,^22^,^23^; a higher copeptin level has been associated with all of these conditions and also predicts outcomes following heart failure and acute myocardial infarction. Previous studies have suggested that a higher copeptin level is an independent prognostic marker for unfavorable functional outcome and mortality in patients with acute ischemic stroke, ICH, and SAH^8^,^9^,^10^,^11^,^12^,^14^,^15^,^16^,^18^. In addition, a high copeptin level has already been successfully validated as independent predictive factor for poor outcome in patients with ischemic stroke especially in the Caucasian population^9^,^11^. These consistent outcomes in various clinical settings relating to plasma copeptin level suggest that strategies for prognostic risk stratification beyond the use of well-known risk factors such as age and stroke severity are necessary for determining early therapeutic interventions, aggressiveness of care, and rehabilitation.

AVP is a potent synergistic factor of the hypothalamo-pituitary-adrenal axis in the production of adrenocorticotropic hormone and cortisol. Previous studies have shown that serum cortisol level is significantly increased proportionately with the degree of stress and may predict outcome in several diseases, including ischemic stroke^24^,^25^. The rationale behind the prognostic role of copeptin in patients with acute stroke is based on its property of mirroring levels of stress more subtly than cortisol^26^. Copeptin is a stable peptide derived from the precursor of vasopressin and is released in an equimolar ratio to AVP^4^. Copeptin has been found to be stable at room temperature and can be easily measured using automated assays, with results available within 20–60 min, promptly enabling the assessment of optimal prognostic stratification^4^,^5^. In addition, it has been assumed that the close and reproducible relationship of copeptin levels to the degree of activation of the stress axis relating to disease severity is the basis of its unique usefulness as a prognostic biomarker^9^. Accordingly, copeptin might have a role as a sensitive surrogate marker for AVP release, indicating the individual stress response.

In contrast to other brain markers, copeptin directly mirrors intracerebral processes and is released into the systemic circulation, thus bypassing the blood-brain barrier^7^. Although the exact mechanism relating copeptin with unfavorable outcome and mortality in acute stroke is not fully understood, brain edema plays a critical role in the pathophysiology and morbidity of a wide variety of nervous system disorders including stroke, infection, and metabolic disorders^27^. In addition, data from experimental studies imply that vasopressin plays a role in brain edema formation and ischemic neuronal injury, as blocking of vasopressin receptors attenuates brain edema in ischemic and traumatic mice models^28^,^29^,^30^,^31^.

The results of our study suggest that the use of a vasopressin antagonist could theoretically be an effective therapeutic strategy for patients with acute stroke. Several studies have suggested that AVP may play a role in the development of ischemic brain edema^32^,^33^. Kozniewska *et al.*^34^ reported that AVP is one of the factors contributing to vasogenic edema and cellular swelling after ICH. According to the large, prospective cohort study by De Marchis *et al.*^9^, space-occupying cerebral edema was associated with higher copeptin values. It is possible that the copeptin level reflects brain edema formation and its severity, thus helping to determine the existence of cerebral edema, which could be treated with an AVP receptor antagonist^20^,^29^. However, there are only a small number of experimental studies that investigate the therapeutic effects of the AVP receptor antagonist for acute stroke. Future trials are therefore necessary to evaluate the efficacy and safety of the AVP receptor antagonist according to stroke subtype.

To the best of our knowledge, this is the first meta-analysis evaluating the prognostic role of copeptin in acute stroke. The findings of this systematic review and collaborative meta-analysis with a total of 2,746 patients demonstrated that plasma copeptin level has prognostic value for the assessment of functional outcome and mortality after acute stroke. In the Caucasian population, copeptin has already been successfully validated as independent prognostic biomarker in patients with acute ischemic stroke, as already shown by two prior independent prospective and large cohort studies^9^,^11^. This meta-analysis confirms, on a larger scale, the validated prognostic role of copeptin in acute ischemic stroke as well as hemorrhagic stroke. Subgroup analysis identified several important findings. A more prominent association was observed between copeptin and mortality after stroke than functional outcome assessment. In addition, subgroup analysis based on the time window of mortality assessment revealed that mortality at 3 months after stroke was more significantly associated with plasma copeptin level than that at 1 year. In the hemorrhagic stroke group, the lower mortality group (<35%) showed a more significant association between copeptin and mortality. Although survival assessment is considered invariable without intra- or inter-observer differences, this finding should be interpreted with caution since the included studies in this meta-analysis did not describe the direct cause of death or the exact time of onset of each complication. Our studies included all patients admitted within a rather large time frame of 72 hours after the onset of clinical symptoms, and the study sample thus constitutes a heterogeneous population. Furthermore, the elevation of plasma copeptin level may indicate that the patient required further evaluation, especially since copeptin is elevated in life-threatening diseases such as shock, renal insufficiency, heart failure, acute myocardial infarction, hospital-acquired pneumonia, and pulmonary thromboembolism^5^,^6^,^8^,^21^,^22^,^23^.

We found significant heterogeneity in the main analysis. Differences in study region may be a possible reason for the heterogeneity, because the I^2^ values were reduced significantly in the stratified analysis according to the study region (the West or China) and significant differences were identified between the two groups. Well-known differences in lifestyle and the incidence, composition, fatality, and mortality of stroke between Western and Eastern countries could have affected the heterogeneity^35^,^36^. Heterogeneity may also result from the different characteristics of the subjects, the various treatment modalities, methodological problems, variable measurement assay for copeptin detection, insensitivity of clinical outcome, and other possible processes contributing to the outcome assessment. However, a stratified analysis according to the different study characteristics did not differ substantially. Moreover, these results were repeated in subgroup analysis of studies from China and Western countries, respectively. These data further support the reliability and stability of the meta-analysis results. Accordingly, we believe that the value of these results should not be underestimated simply because of the overall high heterogeneity, since all of the relevant studies consistently revealed a positive association and low heterogeneity in subgroup analysis between higher copeptin levels and poor outcome after acute stroke.

This meta-analysis has several limitations. First, included studies yielded no data regarding serial measurement of copeptin. Further studies are needed to evaluate whether serial copeptin measurement further improves the risk stratification of acute stroke patients. Second, our meta-analysis failed to identify brain edema formation and link it with copeptin values since imaging studies of the brain were not routinely repeated. Third, publication bias may have been a factor, as negative studies have a lower publication rate and therefore less impact, and the lack of published negative studies in the field of biomarkers with regard to prognosis may have affected the results of the meta-analyses^37^. Finally, our meta-analysis failed to obtain the original data from the assessed studies, which limited further evaluation of the potential roles of copeptin in the assessment of prognostic accuracy in the receiver operating characteristic curve after stroke.

In conclusion, the current systematic review and meta-analysis demonstrates that higher plasma copeptin levels may be an independent prognostic factor for poor functional outcome and mortality in patients with acute stroke. Based on the current findings, early measurement of plasma copeptin could provide better prognostic information for patients with acute stroke and help in decision making for therapeutic interventions. Since potential biases and confounders could not be fully excluded in this meta-analysis, especially in hemorrhagic stroke group or studies from East Asia, well-designed and observational studies with a larger scale are required to confirm this association with underlying pathophysiological mechanisms in the future.

## Methods

We used extensive database searching to find studies that evaluated the prognostic significance of copeptin in acute stroke patients according to the Cochrane review methods^38^.

### Search strategy and data sources

We searched MEDLINE (January 1, 1976 to October 8, 2014), EMBASE (January 1, 1985 to October 8, 2014), the Cochrane Library (January 1, 1987 to October 8, 2014), and KoreaMed (June 1, 1958 to October 8, 2014) without restrictions on language or year of publication. The following keywords were searched: copeptin, neurohypophysis hormone, stroke, brain ischemias, and cerebral hemorrhage. Search strategies were modified for each database using free text terms and controlled vocabularies. The details of search strategies are provided in the Supplementary Fig. S2 (online). We also searched the bibliographies of identified studies and other reviews.

### Study selection

The selection of all studies was independently decided by two reviewers (K.-S.C. and H.-J.C.) according to the predefined selection criteria. Two reviewers screened the titles and abstracts identified from the electronic searches. Full articles of all potentially relevant studies were obtained and assessed. If studies included duplicate data from a previous study, the study with the most up-to-date results was selected. Studies were included in our meta-analysis if they: (1) reported results for patients with acute ischemic stroke (not transient ischemic attack) or hemorrhagic stroke; (2) measured copeptin within the first week after stroke onset; or (3) assessed functional outcome or mortality during follow-up. Hemorrhagic stroke was regarded as spontaneous intracerebral hemorrhage (ICH) or non-traumatic subarachnoid hemorrhage (SAH).

### Data extraction

The study characteristics and results of selected studies were extracted by two independent reviewers. Any disagreement unresolved by discussion was put under the review of the other co-authors (H.-J.Y. and J.-H.C.). The following variables were extracted from studies: first author, year of publication, country, study population, inclusion period, follow-up period, stroke severity according to NIHSS score or Glasgow Coma Scale (GCS) score or World Federation of Neurosurgical Societies (WFNS) grade, assay method for copeptin detection, functional outcome (modified Rankin Scale score), results of survival analysis, odds ratio (OR) or hazard ratio (HR) with 95% confidence interval (CI), and mean copeptin levels with standard deviation (SD); if not available, median values with interquartile range (IQR) were used. If the above variables were not mentioned in the studies, we asked each corresponding author for the data via email.

### Assessment of methodological quality

The methodological quality of identified studies was assessed independently by K.-S.C. and H.-J.C. with blinding to authorship or journal using the Quality in Prognosis Studies (QUIPS) tool, with values of 0, 1, and 2 considered low, unclear, and high, respectively^39^. Studies achieving more than 9 points from the sum of each 6-item score were considered to be high quality. Any unresolved disagreements between reviewers were resolved through discussion or review from the third author. Publication bias was not assessable in these trials. Tests for funnel plot asymmetry are generally only performed when at least 10 studies are included in the meta-analysis. As our analyses for each stroke subtype only included 5 and 8 studies, respectively, tests for asymmetry would be ineffective as they would be unable to differentiate chance from asymmetry.

### Statistical Analysis

In the main analysis, we investigated the association between the initial copeptin level and unfavorable functional outcome/mortality after ischemic or hemorrhagic stroke. We attempted to minimize within-study reporting bias by reporting both studies in which a relative measure of effect (ORs or HRs) and a difference in means was reported. For pooling estimates of the results, we collected ORs or HRs with 95% CIs. Pooled HRs or ORs with 95% CIs were calculated using a random-effects model^40^. Pooled ORs or HRs refer to copeptin levels on a logarithmic scale with base 10. The strength of association of copeptin with death was measured by standardized differences in means (difference in means/pooled SD) between survival and death groups with a random-effects model. Since the unit weights of copeptin levels were expressed as a wide variety of units in the majority of studies, the results were presented as the standardized mean difference (SMD) and 95% CIs to estimate the size of the effect. Copeptin levels across comparison groups were extracted as mean difference ± SD. When the SD was not available in the included studies, we estimated the variance using the following formula^38^: SD = standard error (SE) × sqrt{*N*, sample size}, SD = 1.35/IQR. When both univariate and multivariate results were reported, the latter was used in analysis. To estimate heterogeneity, we estimated the proportion of between-study inconsistency due to true differences between studies (rather than differences due to random error or chance) using the I^2^ statistic, with values of 25%, 50%, and 75% considered low, moderate, and high, respectively^41^.

We conducted planned subgroup analyses based on the type of stroke (hemorrhagic or ischemic); study region; sample size; time window of mortality assessment (within 3 months or within 1 year); case fatality rate (mortality > 35% or lower); statistical assessment method (using HR or OR); and methodological quality of the study (high or low) for the sensitivity analysis. We used RevMan version 5.2 (Cochrane Collaboration, Oxford, UK) for statistical analysis, and *P* < 0.05 was considered statistically significant.

## Additional Information

**How to cite this article**: Choi, K.-S. *et al.* Prognostic role of copeptin after stroke: A systematic review and meta-analysis of observational studies. *Sci. Rep.* **5**, 11665; doi: 10.1038/srep11665 (2015).

## Supplementary Material

Supplementary Information

## Figures and Tables

**Table 1:** **Characteristics of studies included in the review.**

**Study**	**Country**	**Sample size**	**Inclusion period**	**Stroke type**	**Blood collection**	**Copeptin detection method**	**Age, y.**	**Females, %**	**Stroke severity**	**Time of death**	**Death, %**	**Unfavorable outcome, %**
Katan M	Western	359	2006–2007	Ischemic	<72 h	immunoassay(B.R.A.H.M.S)	75 (63–83)	41	5 (2–10)*	3 mo.	12	42
Zweifel C	Western	40	2006–2007	Hemorrhagic	<72 h	immunoassay(B.R.A.H.M.S)	71 (64–78)	45	14 (13–15)**	1 mo.	15	55
Dong XQ	China	86	2006–2008	Hemorrhagic	<24 h	ELISA(Cusabio)	65 (42–80)	23.3	8 (5–13)**	1 wk.	37.2	—
Zhang X	China	89	2007–2009	Hemorrhagic	<24 h	immunoassay(B.R.A.H.M.S)	65 (41–79)	39.3	21 (6–31)*	1 y.	42.7	62.9
Zhang JL	China	245	2007–2010	Ischemic	<72 h	immunoassay(B.R.A.H.M.S)	73 (64–82)	42	6 (3–12)*	1 y.	16.7	40.4
Zhu XD	China	303	2008–2010	Hemorrhagic	<24 h	ELISA(Cusabio)	43.9 ± 12.4	56.7	2.3 ± 1.2***	1 y.	13.9	29.7
De Marchis	Western	783	2009–2011	Ischemic	<24 h	immunoassay(B.R.A.H.M.S)	71 (60–80)	38.1	6 (3–13)*	3 mo.	15.1	38.3
Dong X	China	125	2010–2011	Ischemic	<48 h	immunoassay(B.R.A.H.M.S)	69 (61–85)	44.8	7 (3–12)*	3 mo.	14.4	32.8
Fung C	Western	18	2010–2011	Hemorrhagic	Adm.	immunoassay(B.R.A.H.M.S)	57 (48–67)	66.6	—	6 mo.	22.2	27.7
Tu WJ	China	189	2010–2012	Ischemic	<48 h	immunoassay(B.R.A.H.M.S)	66 (58–75)	38.1	7 (5–12)*	3 mo.	13.2	38.1
Wei ZJ	China	271	2010–2012	Hemorrhagic	<24 h	ELISA(Cusabio)	69 (59–81)	46.9	11 (7–15)**	3 mo.	12.5	30.3
Yu WH	China	118	2010–2013	Hemorrhagic	<6 h	ELISA(Phoenix pharm)	64 (48–79)	39	15 (5–23)*	6 mo.	31.4	57.6
Zhang A	China	120	2013	Hemorrhagic	Adm.	ELISA(Phoenix pharm)	60 (32–84)	37	10.6 ± 4.6**	3 mo.	20	60

Adm., at admission; ELISA, enzyme-linked immunosorbent assay; y, year; mo., month; wk., week; Age and Stroke severity were presented as median (IQR) or mean ± SD.

^*^National Institutes of Health Stroke Scale (NIHSS) score.

^**^Glasgow Coma Scale (GCS) score.

^***^World Federation of Neurosurgeons Societies (WFNS) grade.

**Table 2:** **Summary of estimated odds ratios for functional outcome and mortality among subgroup.**

**Characteristics**	**Functional outcome**				**Mortality**			
	***N***	**OR (95% CI)**	***P*** **value for heterogeneity**	***I***^***2***^**, %**	***N***	**OR (95% CI)**	***P*** **value for heterogeneity**	***I***^***2***^**, %**
Acute stroke								
All	10	1.84 (1.48, 2.29)	<0.0001	82	11	2.66 (1.93, 3.65)	<0.0001	93
					9*	3.61 (2.79, 4.66)	0.13	36
Stroke subtype								
Hemorrhagic	5	1.36 (1.13–1.64)	0.002	77	6	2.16 (1.51, 3.09)	<0.0001	95
					4*	3.66 (2.49, 5.36)	0.09	53
Ischemic	5	2.55 (1.97, 3.31)	0.66	0	5	3.47 (2.38, 5.04)	0.27	23
Study size								
<100	2	3.82 (0.26, 56.96)	0.01	83	3	1.13 (1.03, 1.24)	0.18	42
≥100	8	2.09 (1.55, 2.80)	<0.0001	80	8	3.64 (2.76, 4.80)	0.09	44
Study region								
Western	3	2.66 (1.46, 4.84)	0.16	45	3	2.67 (1.87, 3.80)	0.52	0
China	7	1.63 (1.32, 2.03)	<0.0001	81	8	2.55 (1.80, 3.63)	<0.0001	95
					6*	4.00 (3.00, 5.33)	0.20	31
Number of female								
<40%	4	1.48 (1.15, 1.90)	0.006	76	5	2.09 (1.46, 3.00)	<0.0001	96
					3*	3.79 (2.18, 6.60)	0.02	76
≥40%	6	2.88 (1.55, 5.34)	<0.0001	85	6	3.42 (2.59, 4.52)	0.56	0
Analysis								
Univariate	1	N/A			2	5.02 (3.56, 7.09)	0.39	0
Multivariate	9	N/A			9	2.25 (1.69, 2.99)	<0.0001	91
					7*	3.21 (2.51, 4.11)	0.33	13
Measurement time								
≤24 hour	4	1.51 (1.19, 1.92)	0.0003	84	5	1.70 (1.30, 2.23)	<0.0001	92
					3*	2.79 (2.07, 3.75)	0.27	24
≤72 hour	6	2.61 (1.52, 4.49)	0.0004	78	6	4.85 (3.69, 6.37)	0.89	0
Outcome assessment								
Within 3 month	7	2.40 (1.59, 3.63)	0.0005	75	8	3.08 (1.64, 5.79)	<0.0001	94
					7*	3.71 (2.84, 4.86)	0.20	30
Within 1 year	3	1.34 (1.08, 1.67)	0.004	82	3	2.30 (0.95, 5.57)	<0.001	90
Study quality								
High	8	1.96 (1.52, 2.53)	<0.0001	84	8	2.94 (1.70, 5.08)	<0.0001	91
Low	2	3.95 (0.29, 54.63)	0.02	82	3	2.57 (0.73, 9.00)	<0.0001	97

*N*, the number of studies; OR, odds ratio; 95% CI, 95% confident interval; N/A, not available.

^*^indicate a subgroup analysis after excluding articles which were the key contributors to between-study heterogeneity.

**Table 3:** **Stratified meta-analysis of copeptin levels and poor outcome in patients with acute stroke.**

**Characteristics**	**Functional outcome**				**Mortality**			
	***N***	**SMD (95% CI)**	***P*** **value for heterogeneity**	***I***^***2***^**, %**	***N***	**SMD (95% CI)**	***P*** **value for heterogeneity**	***I***^***2***^**, %**
Acute stroke								
All	12	1.09 (0.81, 1.37)	<0.0001	90	13	1.70 (1.36, 2.03)	<0.0001	87
Stroke subtype								
Hemorrhagic	7	1.25 (0.97–1.53)	0.007	66	8	1.70 (1.18, 2.21)	<0.0001	87
Ischemic	5	0.96 (0.60, 1.32)	<0.0001	90	5	1.67 (1.21, 2.12)	<0.0001	87
Study size								
<100	3	0.75 (–0.01, 1.51)	0.02	73	4	1.27 (0.84, 1.70)	0.17	40
≥100	9	1.16 (0.85, 1.48)	<0.0001	92	9	1.87 (1.46, 2.28)	<0.0001	91
Study region*,**								
Western	4	0.59 (0.42, 0.77)	0.23	30	4	1.18 (1.01, 1.35)	0.54	0
China	8	1.33 (1.09, 1.58)	0.0002	75	9	1.95 (1.56, 2.33)	<0.0001	84
Number of female								
<40%	5	1.21 (0.67, 1.75)	<0.0001	93	6	1.73 (1.26, 2.19)	<0.0001	87
≥40%	7	1.01 (0.66, 1.36)	<0.0001	86	7	1.65 (1.13, 2.17)	<0.0001	88
Method of estimation of the mean and SD**								
Described in the study	4	1.43 (1.23, 1.62)	0.33	13	4	1.76 (1.12, 2.39)	<0.0001	88
Estimated by recognized formulas	8	0.94 (0.60, 1.27)	<0.0001	90	9	1.66 (1.24, 2.09)	<0.0001	88
Measurement time								
≤24 hour	5	1.21 (0.68, 1.74)	<0.0001	94	6	1.62 (1.18, 2.06)	<0.0001	88
≤72 hour	7	1.01 (0.67, 1.35)	<0.0001	82	7	1.75 (1.21, 2.29)	<0.0001	86
Outcome assessment								
Within 3 month	9	1.04 (0.71, 1.38)	<0.0001	90	10	1.70 (1.28, 2.12)	<0.0001	89
Within 1 year	3	1.22 (0.63, 1.80)	<0.0001	90	3	1.68 (1.08, 2.27)	0.001	85
Study quality								
High	9	1.15 (0.83, 1.47)	<0.0001	91	9	1.69 (1.34, 2.05)	<0.0001	88
Low	3	0.79 (–0.08, 1.65)	0.005	81	4	1.61 (0.59, 2.63)	<0.0001	87

*N*, the number of studies; SMD, standardized mean difference; 95% CI, 95% confident interval.

^*^indicate a significant subgroup difference between copeptin level and mortality according to the study region.

^**^indicate a significant subgroup difference between copeptin level and unfavorable outcome according to the study region/method of estimation of the mean and SD.
