# Supplementary material for: Prognostic role of copeptin after stroke: A systematic review and meta-analysis of observational studies
Source: Sci Rep. 2015 Jun 29;5:11665. doi: 10.1038/srep11665 (PMC4483773; doi:10.1038/srep11665)
Supplement: Supplementary Information [file srep11665-s1.pdf]

Prognostic role of copeptin after stroke: A systematic review and meta-analysis of observational studies

Kyu-Sun Choi, Hyun Jung Kim, Hyoung-Joon Chun, Jae Min Kim, Hyeong-Joong Yi,

Jin-Hwan Cheong, Choong-Hyun Kim, Suck-Jun Oh, Yong Ko, Young-Soo Kim,

Koang-Hum Bak, Je-Il Ryu, Wonhee Kim, Taeho Lim, Hyeong sik Ahn, Il Min Ahn,

Seon-Heui Lee

Supplementary Figure 1: Risk of bias and quality assessment of included studies.

	Study Participation	Study Attrition	Prognostic Factor Measurement	Outcome Measurement	Study Confounding	Statistical Analysis and Presentation
De Marchis GM 2013	+	+	+	+	+	+
Dong X 2013	+	?	+	+	+	+
Dong XQ 2011	+	?	?	+	+	−
Fung C 2013	+	?	?	?	−	−
Katan M 2009	+	+	+	+	+	+
Tu WJ 2013	+	?	+	+	+	+
Wei ZJ 2014	+	?	+	+	+	+
Yu WH 2014	+	?	?	+	+	+
Zhang A 2013	?	?	?	?	−	−
Zhang JL 2013	+	+	+	+	+	+
Zhang X 2012	+	?	+	?	+	+
Zhu XD 2011	+	+	+	+	+	+
Zweifel C 2010	+	?	+	+	−	−

Supplementary Figure 2: Comprehensive list presenting the search strategy.

MEDLINE

1. "Brain Ischemias"[tiab] OR "Cerebral Ischemia"[tiab] OR "Cerebral Ischemias"[tiab] OR "Brain Ischemia"[tiab] OR "Brain Infarctions"[tiab] OR "Brain Infarction"[tiab] OR "Posterior Circulation Infarction"[tiab] OR "Anterior Cerebral Circulation"[tiab] OR "Strokes"[tiab] OR "Stroke"[tiab] OR "CVA"[tiab] OR "Cerebrovascular Accident"[tiab] OR "CVAs"[tiab] OR "Cerebrovascular Accidents"[tiab] 176708

2. "Intracranial Hemorrhage"[tiab] OR "Intracranial Hemorrhages"[tiab] OR "Brain Hemorrhage"[tiab] OR "Brain Hemorrhages"[tiab] OR "Cerebral Hemorrhages"[tiab] OR "Cerebral Hemorrhage"[tiab] OR "Intracerebral Hemorrhages"[tiab] OR "Intracerebral Hemorrhage"[tiab] OR "Intracranial Thrombosis"[tiab] OR "Brain Infarctions"[tiab] OR "Brain Infarction"[tiab] OR "Cerebral Hypertensive Hemorrhage"[tiab] OR "Cerebral Hypertensive Hemorrhages"[tiab] OR "Intracranial Hypertensive Hemorrhage"[tiab] OR "Intracranial Hypertensive Hemorrhages"[tiab] OR "SAH"[tiab] OR "Subarachnoid Hemorrhage"[tiab] OR "SAHs"[tiab] OR "Subarachnoid Hemorrhage"[tiab] 34020

3. Brain Ischaemias"[tiab] OR "Cerebral Ischaemia"[tiab] OR "Cerebral Ischaemias"[tiab] OR "Brain Ischaemia"[tiab] 2550

4. "Intracranial Hemorrhages"[Mesh:noexp] OR "Cerebral Hemorrhage"[Mesh:noexp] OR "Brain Infarction"[Mesh] OR "Basal Ganglia Hemorrhage"[Mesh] OR "Intracranial Hemorrhage, Hypertensive"[Mesh] OR "Subarachnoid Hemorrhage"[Mesh:noexp] 72597

5. ("Brain Ischemia"[Mesh]) OR "Stroke"[Mesh] 126994

6. 1-5/OR 270315

7. Glycopeptides[tiab] OR copeptins[tiab] OR "C-terminal provasopressin"[tiab] OR Glycopeptide[tiab] OR copeptin[tiab] 9326

8. ("copeptins" [Supplementary Concept]) OR "Glycopeptides"[Mesh] 48467

9. "Arg-Vasopressin"[tiab] OR "Arg Vasopressin"[tiab] OR "Argipressin"[tiab] OR "Arginine Vasopressin"[tiab] 9827

10. "Arginine Vasopressin"[Mesh]13446

11. 7-10/OR 68962

12. 6 AND 11 398

EMBASE

1. 'Brain Ischemias':ab,ti OR 'Cerebral Ischemia':ab,ti OR 'Cerebral Ischemias':ab,ti OR 'Brain Ischemia':ab,ti OR 'Brain Infarctions':ab,ti OR 'Brain Infarction':ab,ti OR 'Posterior Circulation Infarction':ab,ti OR 'Anterior Cerebral Circulation':ab,ti OR 'Strokes':ab,ti OR 'Stroke':ab,ti OR 'CVA':ab,ti OR 'Cerebrovascular Accident':ab,ti OR 'CVAs':ab,ti OR 'Cerebrovascular Accidents':ab,ti OR 'Brain Ischaemias':ab,ti OR 'Cerebral Ischaemia':ab,ti OR 'Cerebral Ischaemias':ab,ti OR 'Brain Ischaemia':ab,ti 249157

2. 'brain ischemia'/exp OR 'cerebrovascular accident'/exp 273056

3. Brain Ischaemias':ab,ti OR 'Cerebral Ischaemia':ab,ti OR 'Cerebral Ischaemias':ab,ti OR 'Brain Ischaemia':ab,ti 3065

4. 'Intracranial Hemorrhage':ab,ti OR 'Intracranial Hemorrhages':ab,ti OR 'Brain Hemorrhage':ab,ti OR 'Brain Hemorrhages':ab,ti OR 'Cerebral Hemorrhages':ab,ti OR 'Cerebral Hemorrhage':ab,ti OR 'Intracerebral Hemorrhages':ab,ti OR 'Intracerebral Hemorrhage':ab,ti OR 'Intracranial Thrombosis':ab,ti OR 'Brain Infarctions':ab,ti OR 'Brain Infarction':ab,ti OR 'Cerebral Hypertensive Hemorrhage':ab,ti OR 'Cerebral Hypertensive Hemorrhages':ab,ti OR 'Intracranial Hypertensive Hemorrhage':ab,ti OR 'Intracranial Hypertensive Hemorrhages':ab,ti OR 'SAH':ab,ti OR 'Subarachnoid Hemorrhage':ab,ti OR 'SAHs':ab,ti OR 'Subarachnoid Hemorrhage':ab,ti 44930

5. 'brain hemorrhage'/de OR 'brain infarction'/exp OR 'basal ganglion hemorrhage'/exp OR 'brain hemorrhage'/exp OR 'subarachnoid hemorrhage'/de 134142

6. 1-5/OR 430981

7. Glycopeptides:ab,ti OR copeptins:ab,ti OR 'C-terminal provasopressin':ab,ti OR Glycopeptide:ab,ti OR copeptin:ab,ti OR 'Arg-Vasopressin':ab,ti OR 'Arg Vasopressin':ab,ti OR 'Argipressin':ab,ti OR 'Arginine Vasopressin':ab,ti 21203

8. 'copeptin'/exp OR 'glycopeptide'/exp OR 'argipressin'/exp 19472

9. 'neurohypophysis hormone'/de 1310

10. 7-9/OR 29867

11. 6 AND 10 392

COCHRANE

1. "Brain Ischemias" or "Cerebral Ischemia" or "Cerebral Ischemias" or "Brain Ischemia" or "Brain Infarctions" or "Brain Infarction" or "Posterior Circulation Infarction" or "Anterior Cerebral Circulation" or "Strokes" or "Stroke" or "CVA" or "Cerebrovascular Accident" or "CVAs" or "Cerebrovascular Accidents":ti,ab,kw (Word variations have been searched) 25387

2. "Intracranial Hemorrhage" or "Intracranial Hemorrhages" or "Brain Hemorrhage" or "Brain Hemorrhages" or "Cerebral Hemorrhages" or "Cerebral Hemorrhage" or "Intracerebral Hemorrhages" or "Intracerebral Hemorrhage" or "Intracranial Thrombosis" or "Brain Infarctions" or "Brain Infarction" or "Cerebral Hypertensive Hemorrhage" or "Cerebral Hypertensive Hemorrhages" or "Intracranial Hypertensive Hemorrhage" or "Intracranial Hypertensive Hemorrhages" or "SAH" or "Subarachnoid Hemorrhage" or "SAHs" or "Subarachnoid Hemorrhage":ti,ab,kw (Word variations have been searched) 3828

3. "Brain Ischaemias" or "Cerebral Ischaemia" or "Cerebral Ischaemias" or "Brain Ischaemia":ti,ab,kw (Word variations have been searched) 155

4. MeSH descriptor: [Intracranial Hemorrhages] this term only 155

5. MeSH descriptor: [Cerebral Hemorrhage] this term only 661

6. MeSH descriptor: [Brain Infarction] explode all trees 707

7. MeSH descriptor: [Basal Ganglia Hemorrhage] explode all trees 12

8. MeSH descriptor: [Intracranial Hemorrhage, Hypertensive] explode all trees 17

9. MeSH descriptor: [Subarachnoid Hemorrhage] this term only 449

10. #4-9/or 1885

11. MeSH descriptor: [Brain Ischemia] explode all trees 2298

12. MeSH descriptor: [Stroke] explode all trees 5382

13. #11 or #12 6266

14. #1 or #2 or #3 or #10 or #13 28355

15. Glycopeptides or copeptins or "C-terminal provasopressin" or Glycopeptide or copeptin:ti,ab,kw (Word variations have been searched) 225

16. MeSH descriptor: [Glycopeptides] explode all trees 1537

17. "Arg-Vasopressin" or "Arg Vasopressin" or "Argipressin" or "Arginine Vasopressin":ti,ab,kw (Word variations have been searched) 786

18. MeSH descriptor: [Arginine Vasopressin] explode all trees 579

19. #15-18/or 2386

20. #14 and #19 31

KOREAMED

1. "Brain Ischemias"[ALL] OR "Cerebral Ischemia"[ALL] OR "Cerebral Ischemias"[ALL] OR "Brain Ischemia"[ALL] OR "Brain Infarction"[ALL] OR "Stroke"[ALL] OR "CVA"[ALL] OR "Cerebrovascular Accident"[ALL] 3647

2. "Intracranial Hemorrhage"[ALL] OR "Brain Hemorrhage"[ALL] OR "Cerebral Hemorrhage"[ALL] OR "Intracerebral Hemorrhage"[ALL] OR "Intracranial Thrombosis"[ALL] OR "Brain Infarction"[ALL] OR "Cerebral Hypertensive Hemorrhage"[ALL] OR "Cerebral Hypertensive Hemorrhages"[ALL] OR "Intracranial Hypertensive Hemorrhage"[ALL] OR "Intracranial Hypertensive Hemorrhages"[ALL] OR "Subarachnoid Hemorrhage"[ALL] 1775

3. 1 OR 2 5042

4. copeptins[ALL] OR "C-terminal provasopressin"[ALL] OR Glycopeptide[ALL] OR copeptin[ALL] 36

5. "Arg-Vasopressin"[ALL] OR "Arg Vasopressin"[ALL] OR "Argipressin"[ALL] OR "Arginine Vasopressin"[ALL] 129

6. 4 OR 5 165

7. 3 AND 6 3

Supplementary Figure 3: Table presenting the PRISMA guidelines.

Section/topic	#	Checklist item	Reported on page #
**TITLE**			
Title	1	Identify the report as a systematic review, meta-analysis, or both.	1
**ABSTRACT**			
Structured summary	2	Provide a structured summary including, as applicable: background; objectives; data sources; study eligibility criteria, participants, and interventions; study appraisal and synthesis methods; results; limitations; conclusions and implications of key findings; systematic review registration number.	3
**INTRODUCTION**			
Rationale	3	Describe the rationale for the review in the context of what is already known.	5
Objectives	4	Provide an explicit statement of questions being addressed with reference to participants, interventions, comparisons, outcomes, and study design (PICOS).	5
**METHODS**			
Protocol and registration	5	Indicate if a review protocol exists, if and where it can be accessed (e.g., Web address), and, if available, provide registration information including registration number.	
Eligibility criteria	6	Specify study characteristics (e.g., PICOS, length of follow-up) and report characteristics (e.g., years considered, language, publication status) used as criteria for eligibility, giving rationale.	6
Information sources	7	Describe all information sources (e.g., databases with dates of coverage, contact with study authors to identify additional studies) in the search and date last searched.	6
Search	8	Present full electronic search strategy for at least one database, including any limits used, such that it could be repeated.	6
Study selection	9	State the process for selecting studies (i.e., screening, eligibility, included in systematic review, and, if applicable, included in the meta-analysis).	6,8
Data collection process	10	Describe method of data extraction from reports (e.g., piloted forms, independently, in duplicate) and any processes for obtaining and confirming data from investigators.	6,7
Data items	11	List and define all variables for which data were sought (e.g., PICOS, funding sources) and any assumptions and simplifications made.	6,7
Risk of bias in individual studies	12	Describe methods used for assessing risk of bias of individual studies (including specification of whether this was done at the study or outcome level), and how this information is to be used in any data synthesis.	7
Summary measures	13	State the principal summary measures (e.g., risk ratio, difference in means).	7
Synthesis of results	14	Describe the methods of handling data and combining results of studies, if done, including measures of consistency (e.g., I^2) for each meta-analysis.	7,8

Section/topic	#	Checklist item	Reported on page #
Risk of bias across studies	15	Specify any assessment of risk of bias that may affect the cumulative evidence (e.g., publication bias, selective reporting within studies).	7
Additional analyses	16	Describe methods of additional analyses (e.g., sensitivity or subgroup analyses, meta-regression), if done, indicating which were pre-specified.	7,8
**RESULTS**			
Study selection	17	Give numbers of studies screened, assessed for eligibility, and included in the review, with reasons for exclusions at each stage, ideally with a flow diagram.	8
Study characteristics	18	For each study, present characteristics for which data were extracted (e.g., study size, PICOS, follow-up period) and provide the citations.	8
Risk of bias within studies	19	Present data on risk of bias of each study and, if available, any outcome level assessment (see item 12).	8
Results of individual studies	20	For all outcomes considered (benefits or harms), present, for each study: (a) simple summary data for each intervention group (b) effect estimates and confidence intervals, ideally with a forest plot.	8,9
Synthesis of results	21	Present results of each meta-analysis done, including confidence intervals and measures of consistency.	9
Risk of bias across studies	22	Present results of any assessment of risk of bias across studies (see Item 15).	7
Additional analysis	23	Give results of additional analyses, if done (e.g., sensitivity or subgroup analyses, meta-regression [see Item 16]).	9,10,11
**DISCUSSION**			
Summary of evidence	24	Summarize the main findings including the strength of evidence for each main outcome; consider their relevance to key groups (e.g., healthcare providers, users, and policy makers).	11
Limitations	25	Discuss limitations at study and outcome level (e.g., risk of bias), and at review-level (e.g., incomplete retrieval of identified research, reporting bias).	14,15
Conclusions	26	Provide a general interpretation of the results in the context of other evidence, and implications for future research.	15
**FUNDING**			
Funding	27	Describe sources of funding for the systematic review and other support (e.g., supply of data); role of funders for the systematic review.	16

From: Moher D, Liberati A, Tetzlaff J, Altman DG, The PRISMA Group (2009). Preferred Reporting Items for Systematic Reviews and Meta-Analyses: The PRISMA Statement. PLoS Med 6(6): e1000097. doi:10.1371/journal.pmed1000097

For more information, visit: **www.prisma-statement.org**.
